# Dissociating Contributions of the Motor Cortex to Speech Perception and Response Bias by Using Transcranial Magnetic Stimulation

**DOI:** 10.1093/cercor/bhu218

**Published:** 2014-10-01

**Authors:** Eleonore H. M. Smalle, Jack Rogers, Riikka Möttönen

**Affiliations:** 1 Department of Experimental Psychology, University of Oxford, Oxford OX1 3UD, UK; 2 Psychological Sciences Research Institute, Institute of Neuroscience, Université Catholique de Louvain, B-1348 Louvain-la-Neuve, Belgium

**Keywords:** action selection, auditory-motor, categorical perception, sensorimotor, signal detection theory

## Abstract

Recent studies using repetitive transcranial magnetic stimulation (TMS) have demonstrated that disruptions of the articulatory motor cortex impair performance in demanding speech perception tasks. These findings have been interpreted as support for the idea that the motor cortex is critically involved in speech perception. However, the validity of this interpretation has been called into question, because it is unknown whether the TMS-induced disruptions in the motor cortex affect speech perception or rather response bias. In the present TMS study, we addressed this question by using signal detection theory to calculate sensitivity (i.e., *d*′) and response bias (i.e., *criterion c*). We used repetitive TMS to temporarily disrupt the lip or hand representation in the left motor cortex. Participants discriminated pairs of sounds from a “ba”–“da” continuum before TMS, immediately after TMS (i.e., during the period of motor disruption), and after a 30-min break. We found that the sensitivity for between-category pairs was reduced during the disruption of the lip representation. In contrast, disruption of the hand representation temporarily reduced response bias. This double dissociation indicates that the hand motor cortex contributes to response bias during demanding discrimination tasks, whereas the articulatory motor cortex contributes to perception of speech sounds.

## Introduction

It is under debate whether the motor regions that control movements of the articulators during speech production also contribute to speech perception (Scott et al. 2009; Pulvermüller and Fadiga 2010; Hickok et al. 2011a). The key question is whether the articulatory motor cortex is only involved in production of speech sounds or whether it also supports speech perception by generating motor models of speech sounds produced by others (Liberman et al. 1967; Stevens and Halle 1967; Liberman and Mattingly 1985). Transcranial magnetic stimulation (TMS) is a powerful tool to investigate the involvement of the motor cortex in speech perception (Möttönen and Watkins 2012; Möttönen et al. 2014a). Several TMS studies have shown that modulating activity in the primary motor and premotor cortex with TMS can affect performance in speech perception tasks (Meister et al. 2007; D'Ausilio et al. 2009; Möttönen and Watkins 2009; Sato et al. 2009; Bartoli et al. 2015). For example, we have shown that TMS-induced disruption of the lip representation in the primary motor cortex impairs discrimination of synthetic speech sounds that are close to the phonetic category boundary (e.g., “ba” vs. “da”), whereas disruption of the motor hand representation does not affect discrimination performance (Möttönen and Watkins 2009). The findings of this and other TMS studies have been interpreted as support for the idea that the articulatory motor cortex contributes to speech perception, consistent with the weak version of the motor theory of speech perception (Liberman et al. 1967; Liberman and Mattingly 1985) and the concept of analysis by synthesis (Stevens and Halle 1967).

The interpretation that the motor cortex contributes to speech perception has been challenged. It is possible that TMS-induced impairments in performance (i.e., in proportions of correct responses, i.e., “hits”) in speech tasks are caused by disruption of decision making or response selection processes, not perceptual processes. Hickok (2010) has suggested that the TMS-induced disruptions in the motor cortex may have affected response bias, not speech perception, in the above-mentioned TMS studies. Response bias is a tendency to favor one of the response alternatives (e.g., liberal tendency to respond “different” or conservative tendency to respond “same” in a same–different discrimination task), and it can change independently of perceptual sensitivity (Wald 1950; Macmillan and Creelman 2005). Since the previous TMS studies did not dissociate perceptual sensitivity from response bias, it is possible that changes in performance were due to changes in response bias. Indeed, a recent neuroimaging study demonstrates that changes in response bias correlate with wide-spread activity in the fronto-parietal network during discrimination of “ba,” “da,” and “ga” sounds, including regions in the vicinity of the articulatory motor cortex (Venezia et al. 2012). Moreover, it has been shown that user-induced plasticity in the articulatory motor cortex influences response bias during a speech perception task (Sato et al. 2011). These findings suggest that the articulatory motor cortex is involved in postperceptual processes (e.g., response selection and decision making) during speech tasks, but does not necessarily contribute to the perception of speech sounds.

The main aim of the current TMS study was to dissociate the contributions of the motor cortex to speech perception (i.e., sensitivity) and response bias (i.e., criterion) during discrimination of “ba” and “da” sounds. In a previous study we presented pairs of sounds from an acoustic “ba”–“da” continuum with participants indicating whether the sounds were “same” or “different” (Möttönen and Watkins 2009). Since all pairs consisted of acoustically different sounds, we were able to measure proportions of hits (i.e., “different” responses to different pairs) only. In the current study, we also presented pairs of acoustically identical pairs allowing us to also measure proportions of false alarms (i.e., “different” responses to identical pairs), and enabling calculation of sensitivity and response bias. We also included a categorization task in the current study in order to test whether the motor cortex affects categorical perception of “ba”–“da” continuum. In our previous study we found that TMS-induced disruption of the motor lip representation reduced the slopes of category boundaries, but had no effect on their position (Experiment 1 in Möttönen and Watkins 2009). The effect on slopes was not, however, replicated in another experiment (Experiment 2 in Möttönen and Watkins 2009). We applied low-frequency repetitive TMS either over the lip or hand representation of the left motor cortex that induces a temporary disruption in the targeted area (Möttönen and Watkins 2009). The participants performed speech tasks before TMS and immediately after TMS when either the lip or hand representation was still disrupted. The tasks were performed again after a 30-min break, when the motor cortex was recovered from the TMS-induced disruption. We hypothesized that if the articulatory motor cortex contributes to postperceptual processes during syllable discrimination, then TMS-induced disruption in the lip representation should affect response bias. In contrast, if the articulatory motor cortex contributes to speech perception, then TMS-induced disruption of the lip representation should reduce sensitivity (i.e., *d*′). We also investigated the specificity of these effects by disrupting an area outside the articulatory motor cortex (i.e., the hand representation).

## Materials and Methods

### Participants

Twenty-three participants volunteered in the *lip experiment*. Data from 2 participants was unavailable due to technical problems with the testing computer. Data from one participant were excluded from the analyses of both categorization and discrimination tasks because the slope of category boundary in the pre-TMS condition differed by >2 standard deviations from the mean and it was impossible to define between-category pairs. Data from 2 additional participants were excluded from the analyses of the discrimination task due to negative *d*′ values when discriminating between-category pairs (see below). Thus, data from 20 participants were included in the analyses of the categorization task (*n* = 20, 7 males, mean age of 21.85 ± 3.53), and data from 18 participants were included in the analysis of the discrimination task (*n* = 18, 6 males, mean age of 22.0 ± 3.66 SD) in the lip experiment. All participants were native English speakers, except for one participant who was a native German speaker. The latter however spoke English fluently and was exposed to it from the age of 5.

Twenty participants volunteered in the *hand experiment*. Data from one participant were excluded due to discomfort during TMS. Data from 2 additional participants was excluded from the analyses of the discrimination task due to a negative *d*′ value when discriminating between-category pairs (see below). Thus, data from 19 participants were included in the analysis of the categorization task (*n* = 19, 5 males, mean age of 21.9 ± 4.95 SD), and data from 17 participants were included in the analysis of the discrimination task (*n* = 17, 5 males, mean age of 22.2 ± 5.03 SD) in the hand experiment. All participants were native English speakers, except for one participant who was a native Russian speaker. The latter however spoke English fluently and was exposed to English from the age of 3.

Informed consent was obtained from every participant before the start of the experiments. Both experiments were performed under permission from the National Research Ethics Service. All participants were medication-free and had no personal or family history of seizures or other neurological disorders. All participants were right-handed and had normal hearing (self-reported).

### Procedure

**Figure 1.**
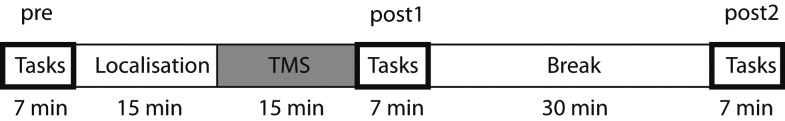
Experimental design. Participants performed identification and discrimination tasks before a 15-min repetitive TMS train (pre), immediately after it (post1) and again after a 30-min break (post2). Before the TMS train the hot spot for either the lip or hand representation in the left motor cortex was localized and the active motor threshold was defined. The TMS-induced disruption lasts for up to 20 min after the end of stimulation. Thus, the motor cortex was expected to recover from the disruption during the break.

Either the lip representation (*lip experiment*) or hand representation (*hand experiment*) in the left M1 cortex were temporarily disrupted by applying a 15-min train of low-frequency repetitive TMS. Participants performed categorization and discrimination tasks before the stimulation (pre), immediately after the stimulation (post 1) and 30 min after post 1 (post 2) (Fig. 1).

### Transcranial Magnetic Stimulation

All TMS pulses were monophasic, generated by a Magstim 200 and delivered through a 70 mm figure-of-eight coil connected through a BiStim module (Magstim) as in our previous studies (Möttönen and Watkins 2009; Möttönen et al. 2013; 2014b). There is evidence that low-frequency trains of monophasic pulses over M1 are more effective in suppressing motor excitability than biphasic pulses (Sommer et al. 2002). The position and angle of the coil over the left motor cortex was adjusted until a reliable motor evoked potential (MEP) was observed in the contralateral lip or hand muscle. Electromyography (EMG) activity was recorded using 2 surface electrodes (22 × 30 mm ABRO neonatal electrocardiogram electrodes) attached to the right corners of the lower and upper lip (orbicularis oris muscle) and from 2 surface electrodes attached to the right hand (first dorsal interosseous muscle), respectively. The ground electrode was attached to the center of forehead. The EMG signals were amplified, bandpass filtered (at 1–1000 Hz) and sampled (at 5000 Hz) using a CED 1902 amplifier, a CED 1401 analog-to-digital converter, and a Windows-PC running Spike software (v. 7; Cambridge Electronic Design).

For each participant the active motor threshold (aMT) was determined: that is, the minimum intensity at which TMS elicited at least 5 out of 10 MEPs at an amplitude of at least 200 μV when the target muscle was contracted at 20%–30% of maximum output. Visual feedback about the level of contraction was provided to the participant to aid him/her to keep this level of contraction. The aMT intensity was used during repetitive transcranial magnetic stimulation (TMS). The mean aMT (percentage of maximum stimulator output, ±SE) for the lip area of left M1 in the lip study was 53.9% (±1.4%). The mean active threshold for the hand area of left M1 in the hand study was 45.5% (±1.9). The aMT for the lip representation is typically higher than the aMT for the hand representation (Möttönen and Watkins 2012; Möttönen et al. 2014a).

During the experiment, 15 min of low-frequency (0.6 Hz) repetitive TMS was delivered over the lip or hand representation of the M1 cortex. Previous studies have shown that 15 min of low-frequency repetitive TMS inhibits excitability of M1 cortex (i.e., reduced MEP amplitudes) for a further 15 min after the end of repetitive (Chen et al. 1997; Möttönen and Watkins 2009). The EMG signal was monitored throughout to ensure that muscles were relaxed and no MEPs were elicited in the target muscle during repetitive TMS. The coil was replaced after 7.5 min to prevent overheating. Insert earplugs were given to participants to protect their hearing. During repetitive TMS the participants watched a nature documentary without sound or subtitles to keep them alert.

### Stimuli

The eight-step phonetic continuum from “ba” to “da” was created using Klatt synthesis (Klatt 1980; for details see Möttönen and Watkins 2009). These synthetic stimuli were created by changing the slope of the formant transition: the onset frequency of F2 was increased from 1100 to 1615 Hz, and that of F3 was increased from 2250 to 2940 Hz in equal steps. The onset frequency of F1 was 400 Hz in all 8 stimuli. The duration of all syllables was 300 ms. The synthetic stimuli were generated to mimic a female voice.

### Tasks

During the categorization task, all 8 stimuli on the “ba”–“da” continuum were presented 12 times in a randomized sequence with a stimulus-onset asynchrony (SOA) of 1500 ms. Participants had to indicate whether they heard “ba” or “da” by pressing the left or right mouse button (2-alternative forced-choice task).

During the discrimination task, participants were presented with pairs of sounds from the /ba/–/da/ continuum. The sounds were either identical (i.e., pairs 1–1, 2–2, 3–3, 4–4, 5–5, 6–6, 7–7, 8–8), or different separated by 2 steps on the continuum (i.e., pairs: 1–3, 2–4, 3–5, 4–6, 5–7, 6–8). The sounds within each pair were presented with a SOA of 500 ms. The same pairs were presented 6 times and the different pairs were presented 12 times (counterbalancing the order of syllables; e.g., 6 times 1–3 and 6 times 3–1, i.e., variable-standard design) in a randomized sequence with a SOA of 2000 ms. The participants were asked to indicate whether the 2 syllables sounded the same or different by pressing the left or right mouse button.

In both tasks, the left mouse button was pressed with the middle finger, and the right mouse button was pressed with the index finger of the left hand. Participants were asked to be as accurate as possible. Presentation software (Neurobehavioral Systems) was used to run the tasks. The stimuli were delivered through high-quality headphones (Sennheiser HD 280 Pro, 64 Ω). The categorization task preceded the discrimination task in all conditions. There was a short break halfway through the discrimination task. During this break the participants were not allowed to speak as this may interfere with the suppressive effect of rTMS.

Before the start of the experiment, the participants were familiarized with the tasks and the stimuli. They first heard a sample of the 2 stimuli at the end-point of the continuum (i.e., stimulus 1 for “ba” and stimulus 8 for “da”). They then practiced the categorization and discrimination task. All stimuli and stimulus pairs were presented during practice. The duration of practice tasks was half of the experimental tasks. Participants were allowed to practice each task twice if necessary.

### Analysis of the Categorization Data

To estimate categorical perception of the “ba”–“da” continuum during the categorization task, logistic curves were fit to each participant's data to obtain slopes and positions of phonetic category boundaries. First, repeated responses (e.g., responding “1” and “2” in rapid succession) and anticipatory responses (i.e.*,* reaction time shorter than 200 ms) were removed from the data. The proportions of “ba” responses were then calculated for all 8 stimuli. The logistic curves were fit to each participant's categorization data in each condition (pre, post1, and post2) using SPSS software (version 19.0), which uses the following formula: *E*(*Y*_t_) = (1 + *β*_0_*β*_1_*^t^*)^−1^. The logarithm of *β*_1_ was used as the slope index. The higher the slope index the steeper the logistic curve (i.e., category boundary). The position of the category boundary was defined as the point along the eight-step continuum corresponding to *E*(0.5).

### Analysis of the Discrimination Data

First, repeated responses (e.g., responding “1” and “2” in rapid succession) and anticipatory responses (i.e., reaction time shorter than 700 ms after the onset the first syllable of the pair) were removed from the data. The stimulus pairs were classified as between-category pairs based on each participant's categorization data as in our earlier study (see Möttönen and Watkins 2009). Between-category pairs were defined as 2 stimuli along the continuum that the participant reliably identifies as belonging to different phonetic categories (i.e., “ba” and “da”). To classify the pairs, the differences between the proportions of “ba” responses to the 2 stimuli in each pair were calculated (i.e., for stimulus pair 3–5: the proportion of “ba” response to stimulus 3 minus the proportion of “ba” response to stimulus 5). If the difference exceeded 0.6, the pair was classified as a between-category pair. For instance, if the proportion of “ba” responses to stimulus 3 was 0.9 and the proportion of “ba” responses to stimulus 5 was 0.3 or less, then the pair 3–5 was classified as a between-category pair (i.e., 0.9 − 0.3 = 0.6). All stimulus pairs that fulfilled this criterion in either the pre- or the post2-condition were selected as between-category pairs. From 1 to 3 stimulus pairs fulfilled this criterion in each participant. On average 1.8 pairs were selected as between-category pairs in the lip experiment and 1.7 pairs in the hand experiment (no significant difference between the experiments). After defining the between-category pairs for each participant, the proportions of “different” responses were calculated for all between-category pairs for each participant. These proportions were then used to calculate hits, defined as the proportion of “different” responses to the different pairs (e.g., 3–5, 5–3, 4–6, and 6–4) and false alarms, defined as the proportion of “different” responses to the corresponding identical pairs (e.g., 3–3, 4–4, 5–5, and 6–6). This was done for every time point (i.e., pre, post1, and post2) separately.

### Calculating Sensitivity (*d*′) and Response Bias (*c*)

Signal Detection Theory provides a means of calculating sensitivity and responses bias based on proportions of hits and false alarms (Wald 1950; Stanislav and Todorov 1999; Macmillan and Creelman 2005). Hits are defined as correctly discriminating stimuli on signal trials, while false alarms are defined as incorrectly discriminating stimuli on noise trials. An internal response continuum determines the threshold for discriminating signal from noise (i.e., *criterion c*). If this criterion is set too low (e.g., due to fatigue) responses will be biased towards detecting signals regardless of actual signal presence, resulting in more hits at the cost of more false alarms (i.e., liberal). If the criterion is set too high (e.g., due to motivation or learning experiences) responses become more conservative (i.e., less likely to detect signals), causing fewer hits and false alarms. The only way to alter hits without also altering false alarms is by altering true perceptual sensitivity, indicated by the normalized distance in means of the Gaussian distributed noise and signal trials (i.e., *d*′). In our previous study, TMS-induced disruption of the lip area caused a decrease in proportion of hits (i.e., “different” responses to pairs of acoustically different sounds, Möttönen and Watkins 2009), but proportion of false alarms (i.e., “different” responses to pairs of acoustically identical sounds) was not investigated. Consequently, it was not possible to calculate sensitivity and response bias and it is not clear whether the changes in hit rate could be explained by changes in response bias (Hickok 2009; Venezia et al. 2012). When response bias changes, the hit rate changes together with the false alarm rate (in the same direction). However, when sensitivity changes, the hit rate changes independently of false alarm rate.

The differencing decision model, appropriate for roving same–different designs, was used to calculate *d*′ and *c* values (see Macmillan and Creelman 2005, Chapter 9, p. 221, for details). We calculated these values using each participant's pooled proportions of “different” and “same” responses for their between-category pairs. Our experiment included a small number of repetitions of each stimulus pair (due to the short duration of TMS-induced motor disruption), and therefore we chose to use pooled proportion rather than calculating *d*′ and *c* values for each pair separately and averaging across (see Macmillan and Creelman 2005, p. 331 for details). The *d*′ *plus* software was used to calculate these values (Macmillan and Creelman 2005; http://psych.utoronto.ca/~creelman). In cases when either the hit rate was 1 or the false alarm rate was 0, these values were adjusted using the method recommended by Macmillan and Creelman (2005, Chapter 1, p. 8).

Positive *d*′ values indicate that the participant was able to distinguish signal from noise, that is, the proportion of hits is higher than that of the false alarms. The higher the *d*′ value the more accurate the discrimination. Negative *d*′ values arise through sampling error or response confusion, resulting in more false alarms than hits (Stanislav and Todorov 1999; Macmillan and Creelman 2005). In the current study, 4 participants showed negative *d*′ values for between-category pairs before the motor disruption (pre). Data from these participants were excluded from the analyses.

### Statistical Analyses

The effects of TMS-induced disruptions on all dependent measures (slopes and positions of category boundaries, hits, false alarms, *d*′, and c) were statistically tested using repeated-measures one-way analysis of variances (ANOVAs) with the factor time (pre, post1, and post2) in the lip and hand experiments separately. Post hoc tests were carried out to test whether pre, post1, and post2 conditions differed significantly from each other using paired *t*-tests (two-tailed).

Specificity of the effects was further investigated by carrying out two-way ANOVAs with experiment (lip vs. hand) as a between-subject factor and time (pre, post1, and post2) as a within-subject factor. Greenhouse–Geisser corrections were used whenever sphericity was violated. Post hoc tests were carried out to compare changes from pre between lip and hand experiments at post1 and post2 time points using independent samples *t*-tests (two-tailed).

## Results

### Categorical Perception of the Stimuli

**Figure 2.**
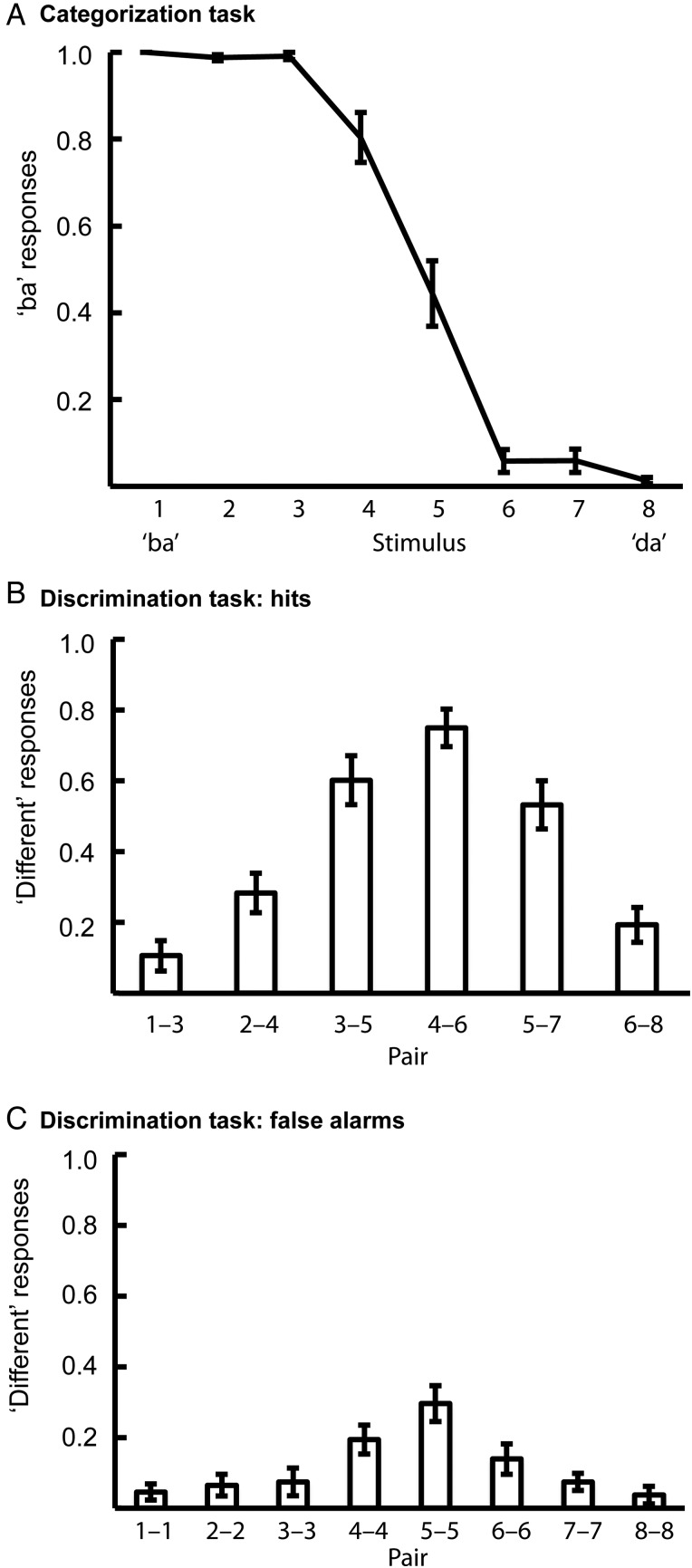
Categorical perception of “ba”–“da” continuum. (*A*) Mean proportions (±SE) of “ba” responses to the eighth-step continuum between “ba” and “da”. (*B*) Mean proportions (±SE) of “different” responses to pairs of acoustically different stimuli (i.e., hits). (*C*) Mean proportions (±SE) of “different” responses to pairs of acoustically identical stimuli (i.e., false alarms). The data are from the pre condition of the lip experiment (*n* = 20). The data from all conditions of both lip and hand experiments are presented in Supplementary Tables 1 and 2.

Participants perceived categorically the 8 stimuli along the “ba”–“da” continuum (Fig. 2, Supplementary Tables 1 and 2). In the categorization task, the participants identified the stimuli 1, 2, and 3 as “ba” and stimuli 6, 7, and 8 as “da” reliably (Fig. 2*A*, Supplementary Table 1). There was a sharp category boundary between stimuli 3 and 6 in each participant, although its position varied across participants. In the discrimination task, the participants gave more “different” responses to the pairs of acoustically different stimuli (i.e., hits) in the middle of the continuum (e.g., 3–5 and 4–6) than to pairs that were close to ends of the continuum (e.g., 1–3 and 6–8; Fig. 2*B*, Supplementary Table 2). This improved discrimination of pairs that consist of stimuli that belong to different phonetic categories (i.e., between-category pairs) relative to pairs of stimuli that belong to the same phonetic category (i.e., within-category pairs) is a hallmark of categorical perception. The participants also gave more “different” responses to the acoustically identical pairs (i.e., false alarms) that were close to the category boundary (e.g., 4–4 and 5–5) than to pairs of stimuli that were far from the category boundary (Fig. 2*C*, Supplementary Table 2).

### Effect of TMS-Induced Disruption on Slopes and Positions of Category Boundaries

**Figure 3.**
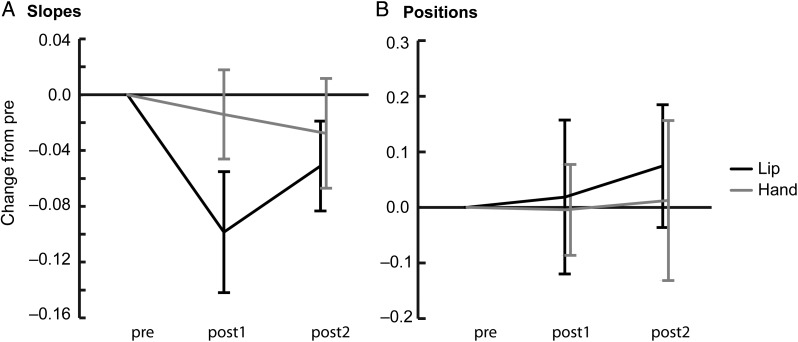
Effects of TMS-induced motor disruptions on slopes and positions of category boundaries. (*A*) Mean changes (±SE) in the slopes of category boundaries in the lip and hand experiments. (*B*) Mean changes (±SE) in the positions of category boundaries in the lip and hand experiments. All changes were calculated relative to the pre condition.

In the lip experiment, the slope of the category boundary changed across time (significant main effect of time: *F*_2,38_ = 3.54, *P* < 0.05, Table 1, Fig. 3*A*). During TMS-induced disruption of the lip area the slope of the category boundary was significantly reduced compared with the pre condition (post1 vs. pre: *t*_19_ = −2.27, *P* < 0.05, Cohen's *d* = 0.51). This reduction in the slope was absent after the break (no significant difference between pre vs. post2). However, the increase in the slope from post1 to post2 was nonsignificant.

**Table 1:** Mean slopes and positions of category boundaries in the lip and hand experiments (±SE)

	Pre	Post1	Post2
Lip experiment			
Slopes	0.97 (0.03)	0.88 (0.05)	0.92 (0.04)
Positions	4.87 (0.11)	4.89 (0.17)	4.95 (0.16)
Hand experiment			
Slopes	0.97 (0.03)	0.96 (0.03)	0.95 (0.04)
Positions	4.68 (0.10)	4.68 (0.09)	4.69 (0.14)

In the hand experiment the slope of the category boundary did not change across time (no significant main effect of time, Table 1, Fig. 3*A*). The change in the slope found in the lip experiment did not, however, differ significantly from the hand experiment (no significant experiment × time interaction).

The position of the category boundary did not change across time in either experiment (no significant main effects of time; Table 1, Fig. 3*B*).

The slope and position of the category boundary did not differ between participants of the lip and hand experiments (no significant main effect of experiment).

### Effects of TMS-Induced Disruptions on Hits and False Alarms

**Figure 4.**
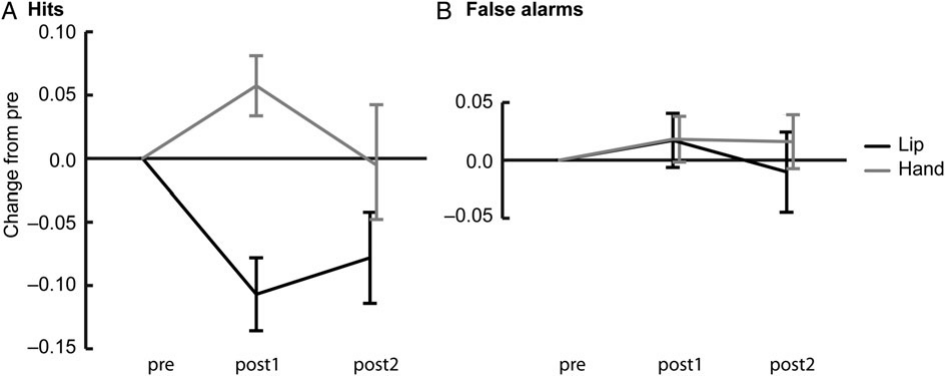
Effects of TMS-induced motor disruptions on discrimination of between-category pairs. (*A*) Mean changes (±SE) in the proportions of hits, that is, “different” responses to acoustically different stimulus pairs in the lip and hand experiments. (*B*) Mean changes (±SE) in the proportions of false alarms, that is, “different” responses to acoustically identical stimulus pairs in the lip and hand experiments. All changes were calculated relative to the pre condition.

In the lip experiment the hit rate for between-category pairs changed significantly across time (*F*_2,34_ = 6.29, *P* < 0.01; Table 2, Fig. 4*A*). During TMS-induced disruption of the lip area the hit rate was significantly decreased compared with the pre condition (post1 vs. pre: *t*_17_ = −3.72, *P* < 0.01, Cohen's *d* = 0.88). The hit rate did not, however, return back to the baseline after the 30-min break (pre vs. post2: *t*_17_ = 2.175, *P* < 0.05; no significant difference between post1 vs. post2, *P* = 0.09).

**Table 2:** Mean proportions of hits and false alarms to between-category pairs in the lip and hand experiments (±SE)

	Pre	Post1	Post2
Lip experiment			
Hits	0.71 (0.04)	0.60 (0.05)	0.63 (0.05)
False alarms	0.17 (0.03)	0.18 (0.03)	0.17 (0.03)
Hand experiment			
Hits	0.69 (0.04)	0.74 (0.04)	0.68 (0.05)
False alarms	0.16 (0.02)	0.18 (0.03)	0.18 (0.03)

The decrease in the hit rate was specific for the lip experiment (significant experiment × time interaction: *F*_1.58, 51.72_ = 6.23, *P* < 0.01, G–G-corrected with *ε* = 0.78; no significant main effect of time in the hand experiment; Table 2, Fig. 4*A*). Specifically, the TMS-induced decrease in hit rate in the lip experiment differed significantly from the change in the hand experiment (post1: lip vs. hand; *t*_33_ = −4.37, *P* < 0.001, Cohen's *d* = 0.23). This difference between the experiments was nonsignificant after the break (i.e., in post2).

The false alarms rates did not change across time in either experiment (no significant main effects of time; Table 2, Fig. 4*B*).

The hit and false alarm rates did not differ between participants of the lip and hand experiment (no significant main effect of experiment).

### Effects of TMS-induced Disruptions on Sensitivity (*d*′)

**Figure 5.**
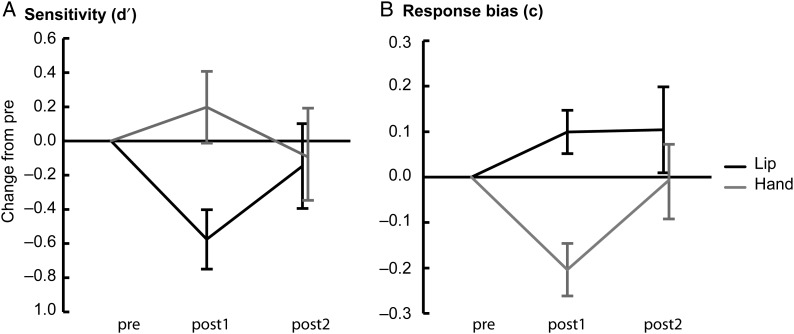
Effects of TMS-induced motor disruptions on sensitive and response bias during discrimination of between-category pairs. (*A*) Mean changes (±SE) in sensitivity (i.e., *d*′) in the lip and hand experiments. (*B*) Mean changes (±SE) in response bias (i.e., *criterion c*) in the lip and hand experiments. All changes were calculated relative to the pre condition.

In the lip experiment the effect of time on sensitivity (*d*′) was marginally significant (*F*_2,34_ = 3.04, *P* = 0.06; Table 3, Fig. 5*A*). During the TMS-induced disruption of the lip area *d*′ was significantly decreased compared with the pre condition (pre vs. post1: *t*_17_ = 2.74, *P* = 0.01, Cohen's *d* = 0.65). This decrease in *d*′ was absent after the break (no significant difference between post2 and pre). However, the increase in *d*′ from post1 to post2 was nonsignificant.

**Table 3:** Mean sensitivity (*d*′) and response bias (*c*) values in the lip and hand experiments (±SE)

	Pre	Post1	Post2
Lip experiment			
*d*′	2.97 (0.20)	2.40 (0.29)	2.83 (0.24)
*c*	0.17 (0.07)	0.27 (0.08)	0.27 (0.11)
Hand experiment			
*d*′	2.98 (0.31)	3.17 (0.27)	2.90 (0.21)
*c*	0.29 (0.08)	0.09 (0.08)	0.28 (0.12)

The decrease in *d*′ was specific for the lip experiment (significant time × experiment interaction: *F*_2,66_ = 3.30, *P* < 0.05; no significant main effect of time in the hand experiment; Table 2, Fig. 3*A*). The TMS-induced decrease in *d*′ in the lip experiment differed significantly from the change in the hand experiment (post1: lip vs. hand: *t*_33_ = −2.82, *P* < 0.01, Cohen's *d* = −0.49). This difference in *d*′ between experiments was absent after the break (i.e., no significant difference in post2).

The *d*′ values did not differ between participants of the lip and hand experiments (no significant main effect of experiment).

### Effects of TMS-Induced Disruptions on Response Bias (*c*)

In the hand experiment the response bias changed significantly across time (*F*_2,32_ = 4.71, *P* < 0.05; Table 3, Fig. 5*B*). During TMS-induced disruption of the hand area the response bias decreased relative to the pre condition (post1 vs. pre: *t*_16_ = −3,53, *P* < 0.01, Cohen's *d* = 0.85). After the break, the response bias returned back to baseline (no significant difference between post 2 and pre; post1 vs. post2, *t*_16_ = 2.36, *P* < 0.05).

The effect of the TMS-induced disruption on the response bias was specific for the hand experiment (significant experiment × time interaction: *F*_1.55, 51.28_ = 4.04, *P* < 0.05, G–G corrected with *ε* = 0.78; no significant main effect of time in the lip experiment). Specifically the TMS-induced decrease in response bias in the hand experiment differed significantly from the change in the lip experiment (post1: lip vs. hand; *t*_33_ = 4.07, *P* < 0.001, Cohen's *d* = 0.71). This difference was absent after the break (i.e., no significant difference in post2).

The *c* values did not differ between participants of the lip and hand experiments (no significant main effect of experiment).

## Discussion

In the current TMS study we investigated contributions of the lip and hand representations in the left motor cortex to speech perception and response bias during a syllable discrimination task. Our results demonstrate a double dissociation. The TMS-induced disruption of the motor lip representation decreased sensitivity (i.e., *d*′) temporarily. In contrast, the TMS-induced disruption of the hand representation had no effect on sensitivity, but decreased the response bias temporarily. This double dissociation indicates that the motor lip representation contributes to perception of speech sounds, whereas the motor hand representation contributes to postperceptual processes.

### Motor Contributions to Speech Perception

Our findings are in agreement with several previous studies showing that TMS over the motor or premotor regions that are involved in speech production affects performance in speech perception tasks (Meister et al. 2007; D'Ausilio et al. 2009; Möttönen and Watkins 2009; Bartoli et al. 2015). Specifically, the decreased hit rate (from pre to post1) found in the lip experiment replicates our previous findings that the TMS-induced disruption of the motor lip representation impairs discrimination of “ba” and “da” sounds (Möttönen and Watkins 2009).

It has been unclear whether the motor cortex contributes to the perceptual or post-perceptual stage of speech processing (e.g., decision-level or response selection processes). The finding that the TMS-induced disruption of the lip representation temporarily decreased sensitivity (i.e., *d*′) provides support for the view that the articulatory motor cortex contributes to perceptual processing of speech sounds. In other words, the difference between “ba” and “da” was less salient during the disruption of the motor lip representation.

The findings are also in agreement with our recent studies showing that TMS-induced disruption in the lip motor cortex affect processing of speech sounds in the auditory cortex (Möttönen et al. 2013; 2014b). In the combined TMS and electroencephalography study we investigated automatic discrimination of speech sounds by measuring mismatch negativity (MMN) responses to changes in sound sequences in the absence of behavioral tasks (Möttönen et al. 2013). The TMS-induced disruption of the motor lip representation suppressed MMN responses to speech sounds, but not to nonspeech piano tones. Furthermore, the TMS-induced disruption of the hand representation had no effect on MMN responses to speech sounds. These findings show that the articulatory motor cortex affects discrimination of speech sounds even in the absence of behavioral speech tasks that require selecting between motor responses (e.g., pressing response buttons). Our combined TMS and magnetoencephalography study showed, however, that articulator-specificity of the motor contributions on auditory speech processing is dependent on behavioral tasks that force listener's to direct their attention on articulatory features of speech sounds (Möttönen et al. 2014b). According to our view the auditory cortex interacts with the articulatory motor cortex during speech processing and these auditory–motor interactions contribute to speech perception. Thus, while the articulatory motor cortex is disrupted, interaction between auditory and motor cortex weakens and efficiency of speech processing reduces. It is, however, possible in principle that TMS over the lip motor cortex stimulates pathways that connect auditory and articulatory motor regions and that the impairments in speech processing after TMS would be due to disruptions in the auditory regions, not in the articulatory motor regions. Speech- and articulator-specificity of the TMS-induced effects on auditory speech processing and their dependence on attention (Möttönen and Watkins 2009; Möttönen et al. 2013, 2014b) lend support for the view that TMS causes a focal disruption in the articulatory motor cortex, which affects speech processing by weakening its interaction with the auditory cortex.

Although the sensitivity was significantly reduced during TMS-induced disruption of the lip representation relative to the pre condition, one-way ANOVA for the lip experiment showed a marginally significant change in sensitivity (main effect of time: *P* = 0.06). Our interpretation that the TMS-induced disruption of the lip representation decreased sensitivity was further supported by the two-way ANOVA that showed a significant interaction between time and experiment. The effect of the disruption on the lip representation on *d*′ differed significantly from the effect of the disruption on the hand representation on *d*′. Also, the finding that the disruption of the lip representation significantly reduced the proportion of hits, but had no effect on false alarms, supports our interpretation that perception of speech sounds was disrupted.

We also tested whether the TMS-induced disruption of the lip area affects the slopes and positions of the category boundary. In our earlier study (Möttönen and Watkins 2009) we found a reduction in the slope of the category boundary between “ba” and “da,” but no change in positions of the category boundaries. These findings were replicated in the current study. The reduction of the slope gives further support for the idea that the articulatory motor cortex contributes to categorical perception of speech sounds. Thus, the slope of the category boundary is shallower, that is, the perception of speech sounds is less categorical, when the articulatory motor cortex is disrupted. The TMS-induced disruption of the hand area had no effect on the slopes in either the current study or our previous study. In the current study, the effect of the TMS-induced disruption of the lip area on the slopes was not, however, as robust as the effect on *d*′, since there were no significant differences between lip and hand experiments in the changes in slopes.

We expected that 15-min of low-frequency repetitive TMS over the motor lip area would disrupt this area for ∼20 min (Möttönen and Watkins 2009). Thus, we predicted that the disruption would be present during post1 condition, but absent during the post2 condition that was started after a 30-min break. In line with this prediction, we found that both *d*′ and slope values were reduced in post1 condition relative to the pre condition, and did not differ significantly between post2 and pre conditions. However, the changes from post1 to post2 were not significant. This suggests that perhaps the motor lip representation did not recover completely from the TMS-induced disruption during the break in all participants. Another plausible explanation is that some participants became tired toward the end of the experiment and, therefore, their performance was not as accurate in the post2 condition as in the pre condition. It is also possible that the TMS-induced disruption of the motor lip area impaired perceptual learning mechanisms (Norris et al. 2003), influencing the perception of speech sounds even after the period of motor disruption had ended and explaining why perception of speech sounds (i.e., *d*′ and the slope of category boundary) did not recover completely during the break.

### Motor Contributions to Response Bias

The TMS-induced disruption of the motor hand representation reduced the response bias (i.e., the criterion) during discrimination of between-category pairs. During the pre condition the participants were rather conservative in selecting “different” responses, that is, they were biased towards “same” responses. This bias was reduced temporarily during the disruption of the hand representation. In principle, this finding is in agreement with the fMRI study of Venezia et al. (2012) that showed that changes in the response bias correlate with the activity in the left-hemisphere fronto-parietal network during discrimination of “ba,” “da,” and “ga” syllables. All participants gave the responses using their left hand in both the current study and that of Venezia et al. (2012). Thus, the response hand was ipsilateral to the TMS-induced disruptions and activity-modulations related to response bias. Venezia et al. manipulated the response bias by changing the proportions of the same and different pairs and found a negative correlation between response bias and activity in several motor areas during syllable discrimination (i.e., the stronger the response bias, the smaller the BOLD signal). This negative correlation is not completely in line with our finding that the disruption of the hand motor cortex decreased the response bias, that is, made participants less biased towards selecting the “same” response. These differences could be due to methodological differences between the studies (e.g., response bias as an independent variable instead of a dependent variable).

It is worth noting that in addition to the decrease in response bias found in the hand experiment of the present study, there was a trend towards an increase in response bias in the lip experiment (see Table 2, Fig. 4*B*). This trend is in agreement with Venezia et al.'s negative correlation and the proposal that the motor regions in the left hemisphere that control the movements of the lips and tongue would also contribute to response bias. Nevertheless, our findings suggest that the contribution of the hand motor cortex to response bias is stronger than that of the articulatory motor cortex, and that these motor regions have opposite effects on response bias. Also, importantly, the articulatory motor cortex contributed to sensitivity, independently of response bias in our study.

The present findings suggest that the hand motor cortex contributes to postperceptual processes such as response selection during a speech discrimination task. Therefore, it is likely that the hand motor cortex should also contribute to response bias in other kinds of tasks, not only in speech tasks. Brasil-Neto et al. (1992) demonstrated that TMS over the hand motor cortex could modulate response bias during a forced-choice task. Also, paired-pulse TMS studies have shown that the dorsal premotor–motor interactions are modulated during response selection (Koch et al. 2006, 2007) and that motor disruptions can delay choice reaction times to visual cues (O'Shea et al. 2007). There is evidence that the left dorsal premotor cortex is involved in selecting actions performed with ipsi- and contralateral hands (Schluter et al. 1998, 2001; Johansen-Berg et al. 2002). It is possible that in the current study TMS over the hand area of the motor cortex also induced a weak disruption in the dorsal premotor cortex. This would explain why the disruptions in the left hemisphere affected response bias when participants used their ipsilateral hand. Further studies should examine whether the TMS-induced disruptions in the left hand motor cortex also modulate response bias when participants use their contralateral hand.

It has been proposed that sensorimotor areas that guide actions (e.g., hand movements) could be functionally involved in perceptual decision-making (Embodied Cognition Theory; Cisek and Kalaska 2010). Recently, this was investigated in an event-related fMRI study in which decisions on house versus face images were performed under varying levels of sensory evidence (Filimon et al. 2013). The perceptual decisions (house vs. face) were decoupled from motor preparation: after the decision period participants were cued to respond with an eye or a hand movement. The authors found evidence for 2 separate systems that implement perceptual and motor decisions. This suggests that the motor areas are important for preparations and indicates that behavioral responses (i.e., hand responses) contribute to motor, but not perceptual, decisions, consistent with the present findings.

### Conclusions

The involvement of the motor cortex in speech perception has been under active investigation in recent years. The key question is whether the human brain generates motor models of the speaker's articulatory movements during listening to speech and whether this process contributes to speech perception. Although numerous studies have shown that the motor cortex is activated during listening to speech (e.g., Fadiga et al. 2002; Watkins et al. 2003; Callan et al. 2004; Wilson et al. 2004; Pulvermüller et al. 2006), the functional significance of such activations is unclear. The patient studies could potentially provide information about the causal role of the motor cortex in speech perception, but their findings have been quite inconsistent. Some studies have shown that patients with frontal brain lesions have impairments in speech perception (Miceli et al. 1980; Baker et al. 1981; Blumstein 1995; Moineau et al. 2005), whereas others have shown that their speech perception can be relatively intact (Hickok et al. 2011b; Rogalsky et al. 2011). TMS provides a tool to investigate the motor contributions to speech perception in the healthy human brain (for a review, see Möttönen and Watkins 2012). The studies using TMS have consistently shown that stimulation of the left-hemisphere motor and premotor cortex changes participants' performance in demanding speech tasks (Meister et al. 2007; D'Ausilio et al. 2009; Möttönen and Watkins 2009; Sato et al. 2009). However, it has been suggested that this change in performance may not necessarily indicate that perception of speech sounds have changed and could also be due to changes at the postperceptual level, for example, during response selection (Hickok 2010). The current TMS study aimed to dissociate the motor contributions to perceptual and postperceptual processes during discrimination of speech sounds. Importantly, the findings show that TMS-induced disruptions in the regions that control the movements of the articulators decrease sensitivity during syllable discrimination, providing strong support for the idea that the articulatory motor cortex contributes to perceptual processing of speech sounds.

## Supplementary Material

Supplementary material can be found at: http://www.cercor.oxfordjournals.org/.

## Funding

This study was funded by the Medical Research Council, UK (career development fellowship to R.M.). E.H.M.S. was supported by the Erasmus placement program (as an MSc student of Ghent University) and J.R. by the Wellcome Trust (WT091070AIA). Funding to pay the Open Access publication charges for this article was provided by the Medical Research Council.

## Supplementary Material

Supplementary Data
