# Supplementary material for: Dissociating Contributions of the Motor Cortex to Speech Perception and Response Bias by Using Transcranial Magnetic Stimulation
Source: Cereb Cortex. 2014 Oct 1;25(10):3690–8. doi: 10.1093/cercor/bhu218 (PMC4585509; doi:10.1093/cercor/bhu218)
Supplement: Supplementary Data [file supp_25_10_3690__index.html]

Dissociating Contributions of the Motor Cortex to Speech Perception and Response Bias by Using Transcranial Magnetic Stimulation — Dissociating Contributions of the Motor Cortex to Speech Perception and Response Bias by Using Transcranial Magnetic Stimulation — Supplementary Data 

# Dissociating Contributions of the Motor Cortex to Speech Perception and Response Bias by Using Transcranial Magnetic Stimulation

## Supplementary Data

Supplementary Data

**Files in this Data Supplement:**

- Supplementary Tables - docx file
