# Supplementary material for: Dissociating Contributions of the Motor Cortex to Speech Perception and Response Bias by Using Transcranial Magnetic Stimulation
Source: Cereb Cortex. 2014 Oct 1;25(10):3690–8. doi: 10.1093/cercor/bhu218 (PMC4585509; doi:10.1093/cercor/bhu218)
Supplement: Supplementary Data [file supp_bhu218_bhu218supp_tables.docx]

**Supplementary Table 1.** Categorization task: mean proportions (%) of ‘ba’ responses to eight stimuli on ‘ba’-‘da’ continuum (±SE).

Pre Post1 Post2

Lip Experiment

Stimulus 1 100.0 (0.0) 97.1 (1.4) 99.2 (0.6)

Stimulus 2 98.8 (0.7) 97.1 (1.3) 97.4 (1.3)

Stimulus 3 99.2 (0.8) 97.9 (0.8) 99.2 (0.6)

Stimulus 4 80.4 (5.8) 80.9 (5.2) 77.5 (6.5)

Stimulus 5 44.4 (7.6) 42.1 (8.0) 37.9 (5.8)

Stimulus 6 5.8 (2.6) 10.0 (3.9) 7.6 (2.1)

Stimulus 7 5,9 (2.7) 5.0 (2.2) 5.4 (2.7)

Stimulus 8 1.3 (0.7) 3.8 (1.5) 5.4 (3.0)

Hand Experiment

Stimulus 1 99.1 (0.6) 99.5 (0.5) 99.1 (0.6)

Stimulus 2 98.6 (0.8) 98.1 (1.1) 98.1 (1.1)

Stimulus 3 99.5 (0.5) 97.6 (1.2) 96.3 (1.2)

Stimulus 4 82.2 (4.5) 77.8 (5.1) 80.1 (5.1)

Stimulus 5 36.4 (6.8) 37.0 (5.7) 38.0 (6.2)

Stimulus 6 3.7 (1.5) 6.5 (2.2) 6.5 (1.9)

Stimulus 7 2.3 (0.9) 1.9 (0.8) 3.2 (1.5)

Stimulus 8 0.5 (0.5) 1.4 (0.8) 0.9 (0.6)

**Supplementary Table 2.** Discrimination task: mean proportions (%) of “different” responses to pairs of identical and different stimuli (±SE).

Pre Post1 Post2

Lip Experiment

*Identical*

1-1 4.6 (2.3) 8.3 (2.4) 7.4 (2.4)

2-2 6.5 (3.1) 4.6 (2.3) 8.3 (2.4)

3-3 7.4 (3.9) 7.4 (3.9) 8.3 (3.6)

4-4 19.4 (4.1) 25.0 (5.1) 25.0 (5.4)

5-5 29.6 (5.1) 31.5 (4.8) 26.5 (3.8)

6-6 13.9 (4.3) 8.3 (2.4) 15.7 (4.4)

7-7 7.4 (2.4) 7. 4 (2.8) 3.7 (2.2)

8-8 3.7 (2.5) 7.4 (3.4) 7.8 (3.5)

*Different*

1-3 10.6 (4.3) 8.1 (2.2) 10.8 (3.6)

2-4 28.3 (5.6) 20.8 (3.7) 19.6 (4.7)

3-5 60.2 (6.9) 50.6 (6.2) 48.5 (7.0)

4-6 75.0 (5.3) 69.9 (5.5) 79.9 (4.3)

5-7 53.2 (6.8) 56.7 (5.6) 58.7 (5.1)

6-8 19.3 (4.9) 26.2 (5.4) 25.6 (5.9)

Hand Experiment

*Identical*

1_1 6.3 (3.1) 4.9 (2.4) 9.8 (5.0)

2_2 5.9 (2.5) 9.8 (3.8) 4.9 (2.4)

3_3 8.0 (2.9) 4.9 (2.4) 10.8 (4.7)

4_4 20.3 (4.1) 29.4 (5.4) 18.6 (5.3)

5_5 23.5 (5.5) 25.5 (5.0) 31.4 (6.0)

6_6 11.8 (3.4) 13.7 (3.3) 8.8 (3.2)

7_7 3.9 (2.3) 2.9 (1.6) 4.9 (2.4)

8_8 9.8 (3.8) 3.1 (1.7) 5.9 (2.5)

*Different*

1_3 10.8 (4.0) 13.8 (4.2) 11.9 (3.8)

2_4 33.4 (6.5) 29.3 (6.0) 34.6 (6.3)

3_5 62.6 (4.5) 63.0 (7.0) 65.5 (5.6)

4_6 66.9 (4.8) 77.9 (5.8) 67.2 (6.5)

5_7 44.6 (6.3) 52.0 (6.9) 47.6 (6.1)

6_8 15.9 (5.9) 15.2 (4.4) 9.3 (3.4)
